# RNA-Binding Proteins in the Post-transcriptional Control of Skeletal Muscle Development, Regeneration and Disease

**DOI:** 10.3389/fcell.2021.738978

**Published:** 2021-09-20

**Authors:** De-Li Shi, Raphaëlle Grifone

**Affiliations:** ^1^Affiliated Hospital of Guangdong Medical University, Zhanjiang, China; ^2^Developmental Biology Laboratory, CNRS-UMR 7622, Institut de Biologie de Paris-Seine, Sorbonne University, Paris, France

**Keywords:** RNA-binding protein, post-transcriptional regulation, myoblast, skeletal myogenesis, muscle regeneration, satellite cell activation, homeostasis, neuromuscular disease

## Abstract

Embryonic myogenesis is a temporally and spatially regulated process that generates skeletal muscle of the trunk and limbs. During this process, mononucleated myoblasts derived from myogenic progenitor cells within the somites undergo proliferation, migration and differentiation to elongate and fuse into multinucleated functional myofibers. Skeletal muscle is the most abundant tissue of the body and has the remarkable ability to self-repair by re-activating the myogenic program in muscle stem cells, known as satellite cells. Post-transcriptional regulation of gene expression mediated by RNA-binding proteins is critically required for muscle development during embryogenesis and for muscle homeostasis in the adult. Differential subcellular localization and activity of RNA-binding proteins orchestrates target gene expression at multiple levels to regulate different steps of myogenesis. Dysfunctions of these post-transcriptional regulators impair muscle development and homeostasis, but also cause defects in motor neurons or the neuromuscular junction, resulting in muscle degeneration and neuromuscular disease. Many RNA-binding proteins, such as members of the muscle blind-like (MBNL) and CUG-BP and ETR-3-like factors (CELF) families, display both overlapping and distinct targets in muscle cells. Thus they function either cooperatively or antagonistically to coordinate myoblast proliferation and differentiation. Evidence is accumulating that the dynamic interplay of their regulatory activity may control the progression of myogenic program as well as stem cell quiescence and activation. Moreover, the role of RNA-binding proteins that regulate post-transcriptional modification in the myogenic program is far less understood as compared with transcription factors involved in myogenic specification and differentiation. Here we review past achievements and recent advances in understanding the functions of RNA-binding proteins during skeletal muscle development, regeneration and disease, with the aim to identify the fundamental questions that are still open for further investigations.

## Introduction

Muscle progenitor cells in vertebrates derive from the paraxial mesoderm located on both sides of the neural tube and the notochord. During early development, the paraxial mesoderm condenses to form segmented somites in an anterior to posterior sequence (McGrew and Pourquié, 1998). The somite undergoes epithelial to mesenchymal transition (EMT) as it matures and further divides into different compartments along dorso-ventral and rostro-caudal axes to form dermatome, myotome and sclerotome, which eventually differentiate into dermis, skeletal muscle, and vertebrae with associated tendons and rib cartilage (Buckingham, 2006). The commitment of progenitor cells to the myogenic lineage and the control of myogenic differentiation depend on the coordinated action of paired box (PAX) transcription factors, like PAX3 and PAX7 (Buckingham and Relaix, 2015), and myogenic regulatory factors (MRFs) of the basic helix-loop-helix (bHLH) family, including MYF5, MYOD, myogenin (MYOG), and MRF4 (Buckingham and Rigby, 2014; Hernández-Hernández et al., 2017; Zammit, 2017). The sine oculis homeodomain (SIX) transcriptional complex, such as SIX1/4, and the bicoid family of homeodomain transcription factor PITX2, also play essential role in this process (Kumar, 2009; Hernandez-Torres et al., 2017; Maire et al., 2020). The PAX3-positive progenitor cells enter into the differentiation program to become MYOD-expressing myoblasts, which proliferate and migrate to their final positions within the body to mature into myogenin-expressing contractile myofibers with syncytial nuclei located at the periphery (Comai and Tajbakhsh, 2014).

Adult skeletal muscle tissue has the remarkable ability to self-repair upon injury by re-activating the myogenic program (Motohashi and Asakura, 2014; Relaix et al., 2021). Although there may be distinct transcriptional regulatory networks in embryonic, fetal, post-natal and adult myogenesis, the process of adult skeletal muscle regeneration recapitulates to a large extent embryonic myogenesis, during which most embryonic muscle regulatory factors are sequentially re-expressed in regenerating muscle fibers with centralized nuclei (Schmidt et al., 2019). This powerful regenerative capacity depends essentially on the activation, proliferation, and differentiation of quiescent muscle stem cells, termed satellite cells (Montarras et al., 2013; Giordani et al., 2018). They are located between the plasma membrane or sarcolemma of each myofiber and its surrounding basal lamina. Their presence within this unique microenvironment or stem cell niche in mature muscles maintains them in a mitotically dormant or quiescent state during tissue homeostasis. These satellite cells are primed for myogenesis with the characteristics of expressing PAX7, so they provide the myogenic precursors that have the capacity of rebuilding functional myofibers to repair the damaged muscle tissue (Chang and Rudnicki, 2014; Dumont et al., 2015; Mashinchian et al., 2018; Ancel et al., 2021).

Thus, critical roles of transcriptional control in the specification, differentiation and regeneration of muscle cells have been well documented in the past decades. At the meantime, the contribution of post-transcriptional regulation to muscle development becomes increasingly important and gains growing interest. RNA-binding proteins (RBPs) are crucial post-transcriptional regulators of gene expression and function in a wide variety of physiological and pathological processes (Glisovic et al., 2008; Ye and Blelloch, 2014; Chen and Hu, 2017). Through temporally and spatially controlled expression, dynamic shuttling between different cellular compartments, and context-dependent interactions with specific partners and mRNA targets, they control RNA metabolism at multiple levels during myogenesis, from pre-mRNA splicing to mRNA transport, localization, stability/degradation, polyadenylation and translation (Apponi et al., 2011; Fujita and Crist, 2018). Mutations or dysfunctions of many RBPs are either directly or indirectly linked to various muscle disorders or neuromuscular diseases in humans, such as myotonic dystrophy (*dystrophia myotonica*) type 1 and 2 (DM1 and DM2), amyotrophic lateral sclerosis (ALS), spinal muscular atrophy (SMA), facioscapulohumeral muscular dystrophy (FSHD), oculopharyngeal muscular dystrophy (OPMD), fragile X syndrome (FXS), to cite a few examples (Brinegar and Cooper, 2016; Conlon and Manley, 2017; Hinkle et al., 2019; Weskamp et al., 2020). Dynamic changes in the expression of RBPs also regulate muscle adaptive processes in response to disuse, aging and exercise (Van Pelt et al., 2019). Functional analyses using cultured cell lines and different vertebrate models have identified increasing numbers of RBPs with conserved and essential functions in embryonic and adult myogenesis. Clearly, cooperative or antagonistic interactions between RBPs in regulating multiple aspects of RNA metabolism tightly coordinate different steps of myogenesis. Thus it is important to gain an overall picture of post-transcriptional circuits exerted by RBPs in muscle physiopathology. The implication of several best-characterized RBPs in muscle development and neuromuscular disease has been discussed in more detail (Apponi et al., 2011; Dasgupta and Ladd, 2012; Raz and Raz, 2014; Hinkle et al., 2019; Nikonova et al., 2019; Sznajder and Swanson, 2019; López-Martínez et al., 2020; Malik and Barmada, 2021). This review attempts to provide a comprehensive outline of studies in understanding functional roles of RBPs during vertebrate embryonic myogenesis and adult skeletal muscle regeneration, with the aim to identify the unanswered questions that merit further investigations.

## An Overview of Post-Transcriptional Regulation in Muscle Development

**FIGURE 1:**
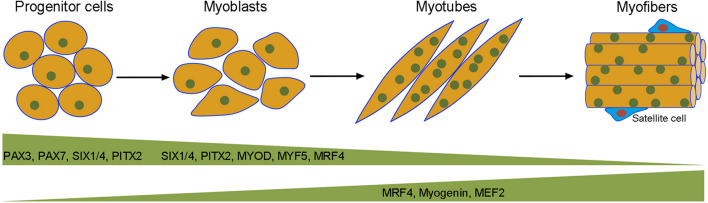
Transcriptional regulation of embryonic myogenesis. Muscle progenitor cells in the somites are committed to the myogenic lineage by the action of PAX3, PAX7, SIX1/4, and PITX2. Mononucleated myoblasts proliferate and differentiate into multinucleated myotubes under the sequential regulation of myogenic transcription factors at different steps of myogenesis. Quiescent satellite cells or muscle stem cells are located between the sarcolemma (plasma membrane) of myofibers and the basal lamina. These cells contribute to muscle regeneration by re-activating the myogenic program.

**FIGURE 2:**
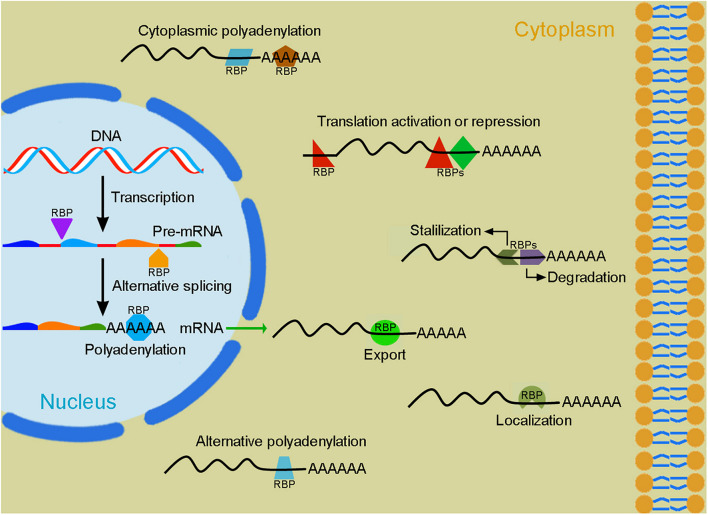
Post-transcriptional regulation of gene expression by RBPs. Following transcription, the dynamic interactions of RBPs (colored forms) with pre-mRNAs or mRNAs in the nucleus and in the cytoplasm regulate their alternative splicing, polyadenylation, export, localization, stability/degradation, and translation. These processes involve a large number of RBPs that function either cooperatively or antagonistically to produce tissue-specific protein isoforms and determine protein levels within a cell.

Myogenesis during pre-natal development and post-natal life is a highly coordinated process controlled by an elaborated interplay of extrinsic and intrinsic regulatory networks (Bentzinger et al., 2012). Proliferating myoblasts derived from progenitor cells in the somites withdraw from the cell cycle and enter into the differentiation program, they eventually fuse with each other to form multinucleated contractile myofibers harboring mitotically quiescent satellite cells in the stem cell niche between the sarcolemma and the basal lamina (Figure 1). Temporally and spatially controlled gene functions are critical for each step of myogenesis. Transcriptional regulation mediated by myogenic transcription factors plays an essential role to commit precursor cells into the myogenic lineage and trigger myoblast terminal differentiation (Buckingham and Rigby, 2014; Hernández-Hernández et al., 2017; Zammit, 2017). However, post-transcriptional regulation of gene expression at multiple levels is also of crucial importance for the progression of myogenesis and the maintenance of muscle homeostasis (Apponi et al., 2011; Hinkle et al., 2019; Nikonova et al., 2019). Throughout myogenesis, proper RNA processing coordinates gene expression and activity required for myoblast proliferation or differentiation (Weskamp et al., 2020). Ultimately, abundant muscle contractile and structural proteins are generated by muscle-specific alternative splicing from genes that normally display wide expression in embryonic or fetal tissues (Llorian and Smith, 2011). Alternative splicing that produces different actin and myosin isoforms also contributes to the development of muscle fiber diversity and the refinement of muscle function (Nikonova et al., 2020). In addition, all stages of myogenesis critically involve the regulation of mRNA stability, localization, polyadenylation and translation to modulate protein synthesis or maintain proteostasis (Figure 2). The interaction between *cis*-regulatory elements present in RNA transcripts and *trans*-acting regulators preferentially expressed in muscle cells tightly controls muscle gene functions and myoblast behaviors at different steps of myogenesis to promote terminal differentiation of functional myofibers.

Thus, post-transcriptional regulation of gene expression orchestrated by RBPs but also other factors, such as non-coding RNAs and even MRFs (Panda et al., 2016; Luo et al., 2021), plays crucial roles in muscle development and regeneration. Many RBPs are differentially associated with target mRNAs in proliferating myoblasts or differentiated myotubes to regulate distinct aspects of RNA metabolism in a stage-dependent manner (Hiller et al., 2020). As will be discussed below, this is often correlated with their dynamic expression and subcellular localization during myogenesis. There are also physical and functional interactions among RBPs in muscle cells, thus they act either cooperatively or antagonistically to determine the activity of target genes for inducing muscle cell proliferation and differentiation or maintaining muscle homeostasis. Mis-regulation in the subcellular accumulation and activity of many RBPs, such as CELF1 and MBNL1, disrupts the expression of muscle-specific protein isoforms and affects either embryonic or adult myogenesis, leading to specific forms of muscular disorders. Altogether, these observations definitely illustrate the importance of RBPs-regulated networks in skeletal muscle physiopathology. Thus, they present the potential to be served as clinically relevant disease-related biomarkers and as therapeutics (Shotwell et al., 2020).

## RNA-Binding Proteins Involved in Skeletal Muscle Development, Regeneration and Disease

### Muscle Blind-Like Proteins Regulate Post-natal Switch of Muscle-Specific Alternative Splicing Patterns

Muscle blind-like (MBNL) protein family members (MBNL1, MBNL2, and MBNL3) are conserved tissue-specific splicing factors involved in the post-transcriptional regulation of gene expression. They recognize YGCY consensus sequence (GC dinucleotides flanked by two pyrimidines) in pre-mRNA and mRNA targets through four highly conserved CCCH-type zinc finger domains (Pascual et al., 2006; Konieczny et al., 2014). Disrupted functions of MBNL proteins, in particular MBNL1 and MBNL2, are associated with DM disease, caused by CUG expansions in the 3′-untranslated region (3′-UTR) of dystrophia myotonica protein kinase gene (*DMPK*) for DM1 or by CCUG expansions in the first intron of cellular nucleic acid-binding protein gene (*CNBP* or *ZNF9*) for DM2. After transcription, these abnormal transcripts form double-stranded hairpins and sequester MBNL proteins into nuclear foci, thus affecting both their cytoplasmic and nuclear pools (Miller et al., 2000) and preventing them from performing normal cellular function as regulators of alternative splicing switch during post-natal development (Pettersson et al., 2015; Sznajder and Swanson, 2019). In addition, likely as an indirect consequence, the massive expansion of toxic CUG repeats also causes abnormal splicing of *MBNL1* and *MBNL2* pre-mRNAs, further adding to changes in the subcellular partitioning and expression of the corresponding proteins in both proliferating myoblasts and differentiating myotubes (André et al., 2019). As a result, multiple aspects of post-transcriptional control are mis-regulated in DM1, including a global disruption of alternative splicing events with abnormal inclusion or exclusion of specific exons as well as defective mRNA localization and translation (Wang et al., 2012).

The three MBNL proteins are differentially expressed during muscle cell differentiation. In C2C12 cells and in mice, MBNL1 protein level remains constant during myogenesis, whereas the expression of MBNL2 and MBNL3 decreases during myogenic differentiation (Holt et al., 2009; Bland et al., 2010). Both MBNL1 and MBNL2 also show translocation from the cytoplasm to the nucleus during myoblast culture and post-natal development (Lin et al., 2006; Holt et al., 2009). This change in subcellular localization may be associated with their activity to switch adult alternative splicing patterns. Functional studies in animal models have provided evidence on the implication of MBNL proteins in muscular dystrophy but also revealed their functional redundancy and specificity in myogenesis. Single knockout or combined loss of *Mbnl* genes in mice disrupts alternative splicing events and polyadenylation patterns, leading to abnormal expression of fetal or non-muscle protein isoforms in adult tissues, which is characteristic of DM disease (Kanadia et al., 2003; Lin et al., 2006; Suenaga et al., 2012; Lee et al., 2013; Batra et al., 2014; Thomas et al., 2017). One of the best-described targets affected by reduced activity of MBNL proteins is the muscle-specific chloride voltage-gated channel 1 gene (*CLCN1*) that functions to regulate muscle contraction and relaxation. Reversion of its fetal splicing pattern in skeletal muscle causes a failure to express the adult isoform of the CLCN1 protein, thus leading to prolonged muscle contractions, which is the hallmark of DM (Charlet-B et al., 2002; Mankodi et al., 2002; Kanadia et al., 2003). Another established target of disrupted MBNL1 function is the bridging integrator-1 gene (*BIN1*) encoding a protein involved in tubular invaginations of muscle membranes and the biogenesis of muscle T tubules, which are specialized membrane structures essential for excitation-contraction coupling. The defective alternative splicing of *BIN1* pre-mRNA in DM1 and DM2 causes muscle weakness due to the expression of an inactive form of BIN1 protein lacking phosphatidylinositol 5-phosphate-binding and membrane-tubulating activities (Fugier et al., 2011). Nevertheless, RNA processing events are also differentially affected by deficiency of individual MBNL proteins (Lin et al., 2006; Poulos et al., 2013), suggesting that they display both redundant and distinct activities in muscle differentiation and function.

Since inhibition of MBNL1 or MBNL2 function disrupts the fetal to adult transition of muscle-specific alternative splicing events, this suggests that they may be required for myogenic differentiation and muscle function. In zebrafish, loss of different *mbnl* genes replicates major features of DM, with altered splicing patterns of muscle function genes, disrupted organization of myofibrils, and reduced amounts of myofibers (Machuca-Tzili et al., 2011; Hinman et al., 2021). By contrast, consistent with its lack of expression in differentiated myofibers and in adult muscle (Lee et al., 2007), MBNL3 promotes myoblast growth and proliferation but inhibits myogenic differentiation. Indeed, constitutive expression of MBNL3 in C2C12 cells prevents myotube formation by suppressing the expression of MYOD, myogenin, and MEF2D (myocyte enhancer factor 2D) through different post-transcriptional mechanisms (Squillace et al., 2002; Lee et al., 2008, 2010), whereas *Mbnl3* knockout myoblasts show defective myogenesis and precocious expression of transcripts associated with terminal differentiation (Poulos et al., 2013; Thomas et al., 2017). Thus MBNL3 normally represses the expression of differentiation-related genes by regulating either pre-mRNA splicing or mRNA levels, an activity that may be partially attributed to its differences in the number of tandem zinc-finger RNA-binding motifs with respect to MBNL1 and MBNL2. In the adult, MBNL3 is transiently expressed during injury-induced skeletal muscle regeneration and is present in activated PAX7-positive satellite cells (Poulos et al., 2013; Thomas et al., 2017). Deletion of the second exon in *Mbnl3* gene, which prevents the expression of the major MBNL3 nuclear isoform, leads to age-dependent delays of muscle regeneration and disrupted muscular function in mice, implying that its mis-regulated expression by toxic CUG repeats may be responsible for the progressive skeletal muscle weakness in DM1 (Poulos et al., 2013).

### CELF1 Functions in Muscle Fiber Type Differentiation

The family of CUG-BP and ETR-3-like factors (CELF) comprises six members (CELF1-6) of structurally related proteins in vertebrates. They possess three RNA recognition motifs (RRMs) that preferentially bind to UG dinucleotide repeats within target transcripts and are involved in multiple aspects of RNA processing, including alternative splicing, RNA editing, deadenylation, mRNA decay and translation (Dasgupta and Ladd, 2012). Most of studies have focused on the function of CELF1, commonly called CUGBP1, because it is also a CUG-binding RBP involved in the pathogenesis of DM (Timchenko et al., 1996; Philips et al., 1998). Unlike MBNL1, CELF1 protein is up-regulated in DM1 myoblasts due to increased stability following hyperphosphorylation by protein kinase C (Lee and Cooper, 2009), but it does not seem to complex with CUG-containing nuclear foci (Timchenko et al., 2001a). CELF1 protein is highly expressed in embryonic and fetal skeletal muscles but dramatically decreases during post-natal development (Brinegar et al., 2017). In C2C12 cells, it is mainly accumulated in the nucleus of undifferentiated myoblasts but is predominantly localized in the cytoplasm of differentiated myotubes (Picchio et al., 2018). The dynamic expression and subcellular localization of CELF1 during skeletal muscle development suggest a role in regulating embryonic splicing patterns of its target transcripts. Consistently, overexpression or increased nuclear expression of CELF1 in adult skeletal muscle leads to changes in alternative splicing events and causes pathological conditions, such as DM and muscle wasting (Ho et al., 2005; Ward et al., 2010; Dasgupta and Ladd, 2012; Cox et al., 2020). Also of note, CELF1 shares both overlapping and distinct RNA targets with MBNL1, but the two proteins function as antagonistic regulators of pre-mRNA splicing and mRNA stability (Ho et al., 2004; Kino et al., 2009; Wang et al., 2015; Brinegar et al., 2017). Thus, gain of CELF1 function and loss of MBNL1 activity collectively lead to the reversion of fetal-specific splicing patterns in adult muscle and contribute to DM pathologies. As an example, overexpressing CELF1 in transgenic mice induces the inclusion of the fetal-specific exon 7a in *Clcn1* transcripts similar to deficiency of MBNL1, both recapitulating the splicing defects in DM (Ho et al., 2005).

CELF1 also regulates the translation of various target mRNAs during muscle development through multiple mechanisms. *Xenopus* CELF1, also known as EDEN-BP, is expressed in the presomitic mesoderm at late stages of development. Inhibition of its activity using morpholinos or neutralizing antibodies impairs somitic segmentation, likely through deadenylation and translational repression of mRNAs encoding Notch pathway proteins (Gautier-Courteille et al., 2004). In contrast to the inhibitory role of increased nuclear CEFL1 on myogenic differentiation, cytoplasmic CELF1 seems to promote myogenesis, although overexpression of CELF1 specifically in the cytoplasm of adult skeletal muscle does not induce muscle defects (Cox et al., 2020). In cultured myoblasts, CELF1 binds to a GC-rich sequence within the 5′ region of *p21* mRNA and induces its translation, thus promoting cell cycle arrest for skeletal muscle differentiation (Timchenko et al., 2001b). In mice, skeletal muscle-specific transgenic overexpression of CELF1 leads to elevated protein levels of MEF2A and p21, which correlates with increased amounts of slow-twitch myofibers and recapitulates alterations of muscle fiber types as well as the muscular dystrophic phenotype of DM1 (Timchenko et al., 2004). Conversely, inhibition of CELF function by muscle-specific expression of a dominant negative CELF protein also causes changes in muscle fiber types and disrupts muscle organization (Berger et al., 2011). Thus, there is a possibility that expression of CELF proteins at the correct time and place during myogenesis regulates the differentiation or proportion of fast and slow myofibers, whereas mis-regulation of CELF1 activity in DM1 myoblasts alters fiber type composition. Since CELF1 is a shuttling protein, it remains to be determined whether its nuclear or cytoplasmic activity, or both, contributes to the development of fiber type diversity. The temporal requirement of CELF proteins for fiber type specification during embryonic myogenesis and the underlying post-transcriptional mechanisms also await further investigations.

### Multiple Heterogeneous Nuclear Ribonucleoproteins Are Involved in Embryonic Myogenesis and Adult Skeletal Muscle Regeneration

Heterogeneous nuclear ribonucleoproteins (hnRNPs) represent a large family of RBPs that share common general features but differ in structural domains, recognition of target sequences, and functional properties. They undergo nucleus to cytoplasm translocation triggered by post-translational modification or by interaction with other hnRNPs, thus their post-transcriptional regulatory functions depend on subcellular localization. Most hnRNPs are predominantly localized in the nucleus of differentiated cells, but they shuttle to the cytoplasm under cellular stress to regulate mRNA turnover and translation. As key proteins involved in RNA metabolism, mutations or dysfunctions of many hnRNPs are linked to various neuromuscular diseases, such as ALS, SMA, and FXS (Geuens et al., 2016), suggesting that they may play important roles in embryonic and adult myogenesis.

One of the major hnRNP, hnRNP D, also known as AU-binding factor 1 (AUF1), comprises four isoforms generated by alternative pre-mRNA splicing. All these isoforms contain two central RRMs and can complex with AU-rich elements (AREs) within the 3′-UTR of target mRNAs to promote their degradation (White et al., 2013). The expression level of AUF1 remains low in adult skeletal muscle but its transcription is activated by CCCTC-binding factor (CTCF) in activated satellite cells during muscle regeneration (Chenette et al., 2016; Abbadi et al., 2019). Consistent with its function as an mRNA decay factor, AUF1 destabilizes several fate-determining checkpoint mRNAs that encode proteins regulating satellite cell proliferation and differentiation, such as twist, cyclin D1, and the sonic hedgehog pathway inhibitor RGS5 (Abbadi et al., 2019). Conversely, in C2C12 myoblasts, it binds to the 3′-UTR of *Mef2C* mRNA and enhances its translation, without affecting its stability; consequently, reducing the expression level of AUF1 delays myogenesis (Panda et al., 2014). Thus, AUF1 promotes muscle development and regeneration by regulating stage-specific targeted degradation of checkpoint mRNAs and by increasing the synthesis of MEF2C protein, a critical regulator of skeletal muscle differentiation (Taylor and Hughes, 2017).

Transactive response DNA binding protein 43 (TDP43 or TARDBP) binds both single stranded DNA and RNA. This hnRNP family protein is predominantly localized in the nucleus but also shuttles to the cytoplasm, thus it may regulate various steps of RNA biogenesis during muscle development and in neuromuscular disease (Picchiarelli and Dupuis, 2020). Mutations in *TARDBP* lead to familial ALS and frontotemporal dementia (FTD), indirectly affecting muscle function (Klim et al., 2021). In zebrafish, knockout of *tardbp* causes muscle degeneration, suggesting that it may be involved in muscle maintenance (Schmid et al., 2013), but the post-transcriptional mechanism remains unclear. C2C12 cells with loss of TARDBP function display impaired myogenesis (Militello et al., 2018; Vogler et al., 2018), and mice lacking one allele of *Tardbp* form smaller myofibers during muscle regeneration, further supporting a requirement of TARDBP for myogenic differentiation and adult skeletal muscle repair (Vogler et al., 2018). In addition, TARDBP can form myo-granules that contain mRNAs encoding sarcomeric proteins and localize to sites of neoforming sarcomeres. These myo-granules are normally cleared in mature myofibers. However, they become abnormally increased in pathological muscle tissues with elevated regeneration, and as a consequence, there is an enhanced formation of amyloid fibers (Vogler et al., 2018). Thus, inappropriate accumulation of myo-granules may lead to the formation of pathological TARDBP aggregates as found in the sarcoplasm of inclusion body myopathy (Salajegheh et al., 2009) and may disrupt muscle structure and function (Tawara et al., 2018).

Guanine-rich RNA sequence binding factor 1 (GRSF1) belongs to the hnRNP F/H subfamily and has high affinity to G-rich RNA sequences susceptible to form G-quadruplexes. It plays an important role in maintaining mitochondrial function. GRSF1 is highly expressed in both differentiating myoblasts and mature muscles (Driscoll et al., 2021). In C2C12 cells, there is evidence that overexpression of GRSF1 inhibits myoblast differentiation by increasing the translational efficiency of mitochondrial *Gpx4* (glutathione peroxidase 4) mRNA through binding to AGGGGA site within the 5′-UTR, while inhibition of GRSF1 promotes myogenesis and accelerates skeletal muscle regeneration probably by preventing GPX4-mediated mitochondrial ROS (reactive oxygen species) elimination (Yin et al., 2019). However, there is also report that loss of GRSF1 does not affect muscle development but reduces the endurance of aged muscles by regulating multiple effector proteins involved in mitochondrial function, inflammation, and ion transport (Driscoll et al., 2021).

Poly(C)-binding protein 1 (PCBP1), also known as hnRNP E1, binds to poly(C) sequence through KH RNA-binding domains and interacts with components of miRNA-processing pathways, such as argonaute 2. It is highly expressed in skeletal muscle during embryonic stages, and inhibition of its function in C2C12 cells promotes myogenesis by modulating the maturation of muscle-enriched miR-1, miR-133, and miR-206, which play a role in skeletal muscle proliferation and differentiation (Espinoza-Lewis et al., 2017). Consistent with an inhibitory role in myogenic specification and differentiation, reduction of PCBP1 expression in mice enhances the differentiation of activated satellite cells into multinucleated myofibers and induces a shift from slow- to fast-twitch myofibers (Espinoza-Lewis et al., 2017).

Polypyrimidine tract binding protein 1 (PTBP1), also known as hnRNP I, is widely expressed and shuttles between the nucleus and the cytoplasm. Its N-terminal region contains a nuclear localization signal and four RRMs that bind UC-rich motifs and mediate homodimer formation (Oberstrass et al., 2005). PTBP1 competes with other RBPs for binding to intronic elements adjacent to regulated exons and acts as a repressor of alternative splicing events. During myogenic differentiation, it antagonizes the activity of RBM4 and CELF1 for switching the myotube-specific inclusion of skeletal muscle exons in α*-tropomyosin* and β*-tropomyosin* mRNAs, respectively (Lin and Tarn, 2005; Sureau et al., 2011). Similarly, PTBP1 represses the inclusion of exon 9 from the capping actin protein of muscle Z-line subunit beta gene (*Capzb*) in opposition to Quaking protein (Hall et al., 2013). These observations suggest that PTBP1 inhibits myogenic differentiation and that overlapping splicing regulatory activities control muscle gene expression during myogenesis.

Several other hnRNPs are also involved in myogenic differentiation or muscle diseases. The N-terminal half of hnRNP A1 contains two RRMs that bind to the UAGGGA/U sequence in target transcripts. Its expression decreases during post-natal development, but increases in regenerating and DM1 skeletal muscles. Overexpression of hnRNP A1 in differentiated myoblasts leads to muscle pathology by antagonizing the activity of MBNL1 and inducing DM1-associated fetal-specific alternative splicing patterns, suggesting that it may function to promote myoblast proliferation and inhibit myogenic differentiation (Li et al., 2020). The functionally versatile hnRNP K contains three KH domains and recognizes poly(C) repeats in target transcripts. As hnRNP A1, its expression is also down-regulated during myogenesis. Knockout of *hnRNP K* in C2C12 myoblasts has been shown to delay cell cycle progression and muscle differentiation by preventing the expression of cell cycle regulators and myogenic factors, raising the possibility that it may be required for myoblast proliferation and differentiation (Xu et al., 2018). The hnRNP L protein contains four RRMs and recognizes CA repeats. Its expression level increases during myogenic differentiation in normal human myoblasts. Knockdown of *hnRNP L* in zebrafish and human cell lines reduces muscle birefringence and impairs myoblast fusion, respectively (Alexander et al., 2021). Moreover, hnRNP L may be also involved in maintaining muscle homeostasis and in modulating DM1 pathologies because it complexes with MBNL1 and forms nuclear protein aggregates in DM1 myoblasts, which partially colocalize with toxic CUG repeats. Bioinformatic analysis reveals that hnRNP L may regulate the expression of multiple target genes that are aberrantly spliced due to loss of MBNL1 activity in DM1 muscle (Alexander et al., 2021), but these *in silico* data need experimental validation.

### Fragile X Mental Retardation Protein and FXR1P Are Critically Implicated in Muscle Development and Disease

The fragile X-related (FXR) family of RBPs include FMRP (fragile X mental retardation protein) encoded by fragile X mental retardation 1 gene (*FMR1*), FXR1P (fragile X-related protein 1) and FXR2P (fragile X-related protein 2) encoded by *FXR1* and *FXR2* genes, respectively (Majumder et al., 2020). They are highly homologous and bind target transcripts through two central KH domains. FMRP and FXR1P also display a C-terminal arginine–glycine–glycine (RGG) box that may be involved in RNA binding as well as homologous and heterologous interactions with other RBPs (Siomi et al., 1994; Winograd and Ceman, 2011). FMRP interacts with argonaute 2 and remodels the ARE complex to activate translation upon serum starvation (Vasudevan and Steitz, 2007). Both FMRP and FXR1P also bind G-quadruplex structures, and they form heterodimer that can increase the dynamics of protein-mRNA interaction (Bechara et al., 2007). Interestingly, the 3′-UTR of *FXR1* mRNA also contains G-quadruplex structures, which stimulate alternative polyadenylation and cause 3′-UTR shortening, thereby preventing microRNA regulation and increasing protein expression (Beaudoin and Perreault, 2013).

In vertebrate embryos, strong expression of *Fmr1* is detected in the developing nervous system, while predominant expression of *Fxr1* and *Fxr2* can be found in the somitic mesoderm (Bourdelas et al., 2004; Tucker et al., 2004). Nevertheless, in contrast to FXR1P, FXR2P shows no or weak expression in adult muscle (Bakker et al., 2000; Huot et al., 2005). Mutations of *FMR1* gene due to unstable expansions of CGG repeats in its non-coding exon cause FXS with mild to moderate intellectual disability, and indirectly lead to muscle pathology (Richter and Zhao, 2021). There is evidence that FMRP is required for maintaining muscle stem cell homeostasis. Loss of its function in mice affects satellite cell differentiation, self-renewal and regeneration to form myofibers. Mechanistically, FMRP regulates the stability and translation of *Myf5* mRNA by releasing the repression of miR-31 (Crist et al., 2012) and by modulating its poly(A) tail length through binding to G-quadruplex structures (Fujita et al., 2017). Thus, these observations suggest a role for FMRP in the repair process of adult skeletal muscle.

Two isoforms of FXR1P (82 and 84 kDa) produced by the incorporation of exon 15 in *FXR1* pre-mRNA are specifically expressed in muscle cells (Khandjian et al., 1998; Dubé et al., 2000). Abnormal splicing of this highly conserved exon 15 in muscle cells is linked to FSHD (Davidovic et al., 2008), although aberrant adult expression of the transcription factor DUX4 is the principal genetic basis of this late onset autosomal dominant muscular dystrophy (Banerji and Zammit, 2021). Recessive mutations in this exon lead to congenital multiminicore myopathy of variable severity in humans and mice, depending on the specific effect of each mutation on FXR1P subcellular localization and function (Estañ et al., 2019). Functional studies suggest that FXR1P is required for muscle development. Blockade of FXR1P activity in *Xenopus* and zebrafish produces muscle-specific effects, resulting in disruption of *MyoD* expression, inhibition of somitic myotomal cell rotation and segmentation, and abnormal dermatome formation (Huot et al., 2005; Van’t Padje et al., 2009). Loss of FXR1P in mice leads to a reduction of limb musculature and a decreased amount of contractile filaments in skeletal muscle (Mientjes et al., 2004). The post-transcriptional mechanisms underlying FXR1P regulatory functions in muscle development need further investigations. However, there is evidence that FXR1P promotes myogenic differentiation in C2C12 cells and in human myoblasts by regulating the stability of *p21* mRNA, thus its loss of function causes an up-regulation of p21 activity and induces a premature cell-cycle arrest that impairs myogenesis (Davidovic et al., 2013). Recently, It has been shown that the developmentally regulated muscle-specific inclusion of exon 15 in *Fxr1* mRNA is required for the formation of biomolecular condensates in differentiating C2C12 myoblasts and for somite organization in *Xenopus* embryos (Smith et al., 2020). The property of FXR1P to undergo liquid–liquid phase separation is dependent on its intrinsically disordered domain (IDD) encoded by exon 15 and on its RNA-binding function. It is possible that these liquid-like assemblies may pattern the developing muscle by modulating FXR1P interaction with other RBPs or mRNA targets (Smith et al., 2020). Contrary to FXR1P, there is at present no functional evidence of FXR2P in muscle development.

### RNA Binding Forkhead Box Homolog Family Proteins Regulate Muscle-Enriched Alternative Splicing Patterns

The RNA binding forkhead box homolog (RBFOX) family of RBPs include three members in vertebrates (RBFOX1, RBFOX2, and RBFOX3). They contain an evolutionarily conserved RRM that binds to UGCAUG motif at regulatory sites in pre-mRNAs and mRNAs with high affinity and specificity (Conboy, 2017). At present, no skeletal muscle disease has been directly associated with mutations of *RBFOX* genes. However, in an FSHD mouse model and in FSHD patients, the down-regulated expression of RBFOX1 protein due to decreased mRNA stability causes defective splicing of calpain 3 pre-mRNA, which generates a protein isoform that disrupts the balance between global protein synthesis and degradation in adult muscle (Pistoni et al., 2013). In C2C12 cells, RBFOX1 expression is up-regulated in differentiated myotubes, while RBFOX2 (also known as RBM9) expression level remains constant during myogenesis (Bland et al., 2010). This suggests that they may be distinctly involved in the post-transcriptional regulation of myoblast proliferation and differentiation. Functional analyses show that both proteins are required for myoblast fusion during C2C12 differentiation by binding to *Mef2D* pre-mRNA and promoting its muscle-specific alternative splicing (Singh et al., 2014; Runfola et al., 2015). In mice, conditional knockout of *Rbfox1* gene in skeletal muscle impairs muscular function but not skeletal muscle regeneration, by disrupting alternative splicing of multiple genes encoding proteins involved in myofibril structure, cytoskeletal organization, and calcium signaling (Pedrotti et al., 2015). Combined loss of *Rbfox1* and *Rbfox2* genes in adult mouse skeletal muscle disrupts a large number of alternative splicing events, resulting in the absence of muscle-specific isoforms of MEF2A and MEF2D, which are required for late stages of muscle differentiation, but also leading to increased expression of an active form of the calpain 3 protease that alters proteostasis in muscle cells. As a result, double knockout mice show severe loss of skeletal muscle mass and strength, suggesting an important role for these two RBPs in maintaining muscle homeostasis (Singh et al., 2018). At present, no muscle developmental role has been ascribed to RBFOX3, also known as NEUN (NEUronal Nuclei), likely because it mainly functions in neuronal differentiation.

The function of RBFOX proteins in muscle physiology is also conserved in other vertebrates. In zebrafish, there are five *rbfox* paralogs (*rbfox1*, *rbfox1l*, *rbfox2*, *rbfox3a*, and *rbfox3b*). Each *rbfox* gene can generate multiple splice variants^1^. During early development, *rbfox1l* is expressed in adaxial somitic mesodermal cells corresponding to the precursors of slow-twitch muscle fibers, while *rbfox2* shows more broad expression in the presomitic mesoderm and in the somites (Gallagher et al., 2011). Individual knockdown of *rbfox1l* or *rbfox2* does not affect muscle development; however, *rbfox1l* and *rbfox2* double morphants show alterations of a subset of muscle-specific splicing events and display defective myofibril assembly, suggesting that the Rbfox regulatory network is required for the expression of genes with important muscular functions (Gallagher et al., 2011). In zebrafish adult muscle, Rbfox1l and Rbfox2 also exhibit partial overlapping and distinct expression patterns in Pax7-positive satellite-like cells. Both proteins are rapidly up-regulated in satellite-like cells and in neoforming myofibers during skeletal muscle regeneration, with the expression of Rbfox2 preceding that of Rbfox1l (Berberoglu et al., 2017). These observations imply that Rbfox1 may promote myogenic differentiation, while Rbfox2 may function to regulate satellite cell specification and maintenance in zebrafish. However, there is at present no direct functional evidence regarding their requirement for the development of fiber types and for the muscle repair process.

### STAU1 Inhibits Embryonic Myogenesis and Maintains Satellite Cell Quiescence

Vertebrate Staufen proteins (STAU1 and STAU2) contain four double-stranded RNA-binding domains and play an important role in regulating mRNA localization and stability through a STAU1-mediated mRNA decay (SMD) mechanism (Miki et al., 2005). STAU1 has complex roles in the pathology of DM1 and is associated with muscle atrophy by interfering with multiple post-transcriptional processes. In transgenic mice, sustained expression of STAU1 in post-natal skeletal muscle causes a myopathy phenotype by increasing the expression of phosphatase tensin homolog (PTEN) and by inhibiting phosphoinositide-3-kinase (PI3K)/AKT signaling (Crawford Parks et al., 2017). Further overexpression of STAU1 in a mouse model of DM1 exacerbates the myopathy phenotype through a similar mechanism, suggesting that STAU1 is an atrophy-associated gene with impact on progressive muscle wasting in DM1 (Crawford Parks et al., 2020). However, there is also evidence indicating that increased expression of STAU1 may have a beneficial effect on DM1. Likely representing an adaptive response to muscle pathology, the expression of STAU1 is strongly increased in DM1 muscle cells where it interacts with CUG-expanded mutant mRNAs, resulting in their enhanced nuclear export. As a consequence, this may mitigate the combined negative consequences of reduced MBNL1 function and increased CELF1 activity on the reversion of fetal splicing patterns in adult muscle (Ravel-Chapuis et al., 2012). Further studies indicate that STAU1 exerts broad effects on alternative splicing events that have either positive or negative impact on the DM1 pathology, thus it may function as a disease modifier with both beneficial and detrimental outcomes (Bondy-Chorney et al., 2016).

STAU1 protein highly accumulates in the cytoplasm of proliferating myoblasts during early stages of myogenic differentiation and muscle regeneration but is present at a low level in the nucleus of mature muscles (Ravel-Chapuis et al., 2014). Knockdown of *Stau1* in C2C12 cells triggers myogenic differentiation independently of SMD, but the exact mechanism remains unclear (Yamaguchi et al., 2008). Conversely, overexpression of STAU1 prevents myogenesis by promoting the translation of *c-myc* mRNA (Ravel-Chapuis et al., 2014). These observations seem to suggest that STAU1 functions to inhibit myogenic differentiation. Consistently, in muscle satellite cells, STAU1 has been shown to mediate stem cell quiescence through translational repression of *MyoD* mRNA by binding to its 3′-UTR, thereby preventing cell cycle entry and satellite cell activation, while reducing the expression level of STAU1 leads to increased MYOD protein synthesis and promotes myoblast proliferation (de Morrée et al., 2017). Thus, STAU1 regulates embryonic and adult myogenesis through translational activation and repression of distinct mRNAs. Functional implication of STAU2 in muscle development has not been demonstrated at present. Nevertheless, it has been shown that both STAU1 and STAU2 preferentially localize to the neuromuscular junction and are expressed at higher levels in slow-twitch muscles. Their expression also increases in C2C12 myoblasts during differentiation and in adult muscle following denervation (Bélanger et al., 2003).

### Nuclear Poly(A)-Binding Protein 1 Regulates Poly(A) Tail Length and Polyadenylation Site Utilization in Muscle Cells

Nuclear poly(A)-binding protein 1 (PABPN1) functions in the nuclear cleavage and polyadenylation complex. It is an abundant nuclear protein that plays an essential role in mRNA polyadenylation (Charlesworth et al., 2013). The N-terminal domain of PABPN1 contains a stretch of 10 alanine residues and a coiled–coiled region that is required for binding to poly(A) polymerase. These are followed by a single RRM involved in polyadenylated mRNA binding and PABPN1 oligomerization. The C-terminal region contains a nuclear localization signal and also contributes to PABPN1 oligomerization (Banerjee et al., 2013). A short expansion of the polyalanine tract in PABPN1 leads to a mis-folded protein prone to form nuclear insoluble aggregates and is considered as the main cause of OPMD, a late onset autosomal dominant myopathy (Brais et al., 1998; Vest et al., 2017). The expression of both *PABPN1* mRNA and PABPN1 protein becomes decreased in OPMD-affected muscles, but is increased after muscle injury, suggesting that it may play a role in muscle regeneration and homeostasis (Apponi et al., 2013). Indeed, inhibition of PABPN1 function in primary mouse myoblasts derived from extraocular, pharyngeal and limb muscles impairs proliferation and differentiation by inducing a general shortening of mRNA poly(A) tail length and by preventing the export of polyadenylated mRNAs from the nucleus, thus altering gene expression during myogenesis (Apponi et al., 2010). Moreover, reduced PABPN1 levels in mouse adult skeletal muscle affect polyadenylation site utilization within the 3′-UTR of several mRNAs associated with OPMD, resulting in the usage of more proximal alternative polyadenylation sites (de Klerk et al., 2012; Abbassi-Daloii et al., 2017; Raz et al., 2017). For examples, this causes increased expression of the muscle atrophy regulator atrogin-1, and impairs cytoskeletal organization, sarcomeric protein expression, and myogenic differentiation, leading to muscle pathology (Riaz et al., 2016; Olie et al., 2020). PABPN1 also functions with other proteins in regenerating skeletal muscle and in differentiating C2C12 cells. In particular, it interacts with matrin3, an RNA- and DNA-binding nuclear matrix protein whose mutations are associated with a rare late-onset distal myopathy and ALS (Iradi et al., 2018; Malik and Barmada, 2021). The two proteins colocalize in discrete nuclear foci and promote myogenesis by regulating polyadenylation site selection in myogenic transcripts (Banerjee et al., 2017).

### Human Antigen R Regulates mRNA Stability to Promote Myogenic Differentiation

Human antigen R (HuR) or ELAV-like protein 1 (ELAVL1) is a ubiquitously expressed protein that belongs to the ELAV (embryonic lethal abnormal vision in *Drosophila*) family. It contains three RNA-binding domains and recognizes AREs within the 3′-UTR of mRNAs (Ma et al., 1996). During myogenic differentiation, this protein shuttles from the nucleus to the cytoplasm through the HuR nuclear-cytoplasmic shuttling (HNS) motif and regulates the expression of several myogenic factors by stabilizing their mRNAs (von Roretz et al., 2011). Thus, the nucleus to cytoplasm translocation of HuR is critical for myogenesis. This is achieved by caspase-dependent cleavage of the full-length HuR to generate HuR-CP1 and HuR-CP2 fragments. HuR-CP1 expression level increases during myoblast differentiation and complexes with the HuR import factor transportin-2 (TRN2) to prevent TRN2-mediated import of the full-length HuR into the nucleus, thereby allowing HuR to promote myogenesis by stabilizing muscle-specific mRNAs in the cytoplasm (van der Giessen and Gallouzi, 2007; Beauchamp et al., 2010). At the onset of myogenic differentiation in C2C12 cells and at early stages of skeletal muscle regeneration in mice, HuR is abundantly localized to the cytoplasm, while it returns to the nucleus in differentiated myotubes (Figueroa et al., 2003). The cytoplasmic accumulation of HuR in differentiating myoblasts promotes the fusion of myoblasts to form myotubes by increasing the expression and half-life of several mRNAs encoding regulators of cell cycle withdrawal and muscle differentiation or function, such as p21, MYOD, myogenin, and acetylcholinesterase (Figueroa et al., 2003; van der Giessen et al., 2003; Deschênes-Furry et al., 2005; Beauchamp et al., 2010). These mRNAs are normally degraded in proliferating myoblasts by K-homology (KH)-type splicing regulatory protein (KSRP), an ARE-directed mRNA decay-promoting RBP (Briata et al., 2005). Thus, the coordinated activity of HuR and KSRP regulates the progression of myoblast differentiation. Conversely, HuR forms a complex with KSRP to destabilize nucleophosmin mRNA, which encodes a regulator of cell cycle progression, thus promoting early steps of myogenesis in C2C12 cells (Cammas et al., 2014).

Human Antigen R also interacts with non-coding RNAs to regulate the stability and translation of its target mRNAs. In C2C12 cells, it binds to and enhances the translation of *Hmgb1* mRNA by preventing the translational repressive activity of miR-1192, thereby promoting the commitment of myoblasts into the myogenic program (Dormoy-Raclet et al., 2013). In human myoblasts, HuR triggers myogenic differentiation by stabilizing *MEF2C* mRNA with the aid of IncRNA OIP5-AS1 (Yang et al., 2020). However, under inflammation-induced muscle wasting of C2C12 myotubes, HuR activates the translation of *Stat3* (signal transducer and activator of transcription 3) mRNA and acts as an inducer of muscle loss by preventing the inhibitory effect of miR-330 on *Stat3* mRNA translation (Mubaid et al., 2019).

Constitutive knockout of *HuR* gene in mice is embryonic lethal due to defects in extraembryonic placenta, but *HuR*-null embryos rescued from the placental failure display reduced limb size and impaired endochondral ossification (Katsanou et al., 2009). Although the developmental gene programs affected by loss of HuR function are complex, it seems that only mRNA stability is affected, either positively or negatively. For example, embryonic fibroblasts derived from *HuR*-null mice show a decreased half-life of *Ets2*, *Hoxd13*, and *Tbx4* mRNAs, which encode transcription factors involved in limb patterning, identity and outgrowth, but they also exhibit an increased expression of *Hoxb9* mRNA encoding a homeodomain protein that participates in skeletal development, suggesting a role of HuR in orchestrating gene expression programs for skeletal specification patterns (Katsanou et al., 2009). Unexpectedly, conditional knockout of *HuR* gene in mouse skeletal muscle does not cause muscular defects; however, this induces an increased proportion of oxidative type I fibers, thus enhancing exercise endurance by increasing the steady-state level of *Ppargc1a* (peroxisome proliferator-activated receptor-gamma coactivator-1α) mRNA (Janice Sánchez et al., 2019). Since PPARGC1A, also called PGC1α, functions as a key regulator of cellular energy metabolism, this observation suggests that HuR may regulate the formation or maintenance of type II fibers in adult skeletal muscle by inhibiting the expression of PPARGC1A. Indeed, HuR acts together with KSRP to destabilize *Ppargc1a* mRNA in muscle cells (Janice Sánchez et al., 2019). Thus, HuR may be involved in muscle development and function by regulating the stability of its mRNA targets in a context-dependent manner.

### RNA Binding Motif Protein 24 Is Required for Embryonic Myogenesis and Adult Skeletal Muscle Regeneration

RNA binding motif protein 24 (RBM24) contains a single RRM at the N-terminus that is highly conserved from *Caenorhabditis elegans* to humans and binds to GU-rich sequences in target transcripts (Grifone et al., 2020). In all vertebrate species, it is strongly expressed in the paraxial mesoderm and in the heart, as well as in differentiating cells of various head sensory organs (Grifone et al., 2014, 2018). Moreover, RBM24 protein displays characteristic cytoplasm to nucleus translocation during muscle cell development. In the early myotome of mouse embryos at E11.5 stage of development, it is accumulated in the cytoplasm of MYOD-expressing myoblasts, but is absent in dermomyotomal and migrating PAX3-positive premyogenic progenitor cells (Grifone et al., 2014). However, it becomes restricted in periphery nuclei of adult skeletal muscle fibers but is not expressed in satellite cells (Grifone et al., 2021). This translocation from the cytoplasm of myoblasts to the nucleus of myotubes is also evident during differentiation of C2C12 cells, suggesting dynamic functions of this RBP in myogenesis (Grifone et al., 2021). Consistently, *Xenopus Rbm24* (also known as *Xseb4*) gene is a direct transcriptional target of MYOD and early B cell factor (EBF), suggesting that it functions in the differentiation step of myogenesis (Li et al., 2010; Green and Vetter, 2011). This regulatory hierarchy is in agreement with the binding of MYOD to *Rbm24* genomic sequence (Maguire et al., 2012), and is at least partially conserved in other vertebrates. Indeed, *Rbm24* expression in homozygous *MyoD* mutant mice is completely lost in head muscles and strongly reduced in limb muscles, but seems to be unaffected in the myotomes (Grifone et al., 2014). The zebrafish genome harbors two *rbm24* paralogs, *rbm24a* and *rbm24b*, which show highly overlapping expression patterns in the somites. Knockdown of either *rbm24a* or *rbm24b* impairs somitogenesis by inducing the expression of aberrantly spliced isoforms of Notch pathway ligands (Maragh et al., 2014). In chick embryos, inhibition of RBM24 function in the somites severely reduces the expression of muscle-specific myosin and impairs myogenic differentiation (Grifone et al., 2014). Nevertheless, the post-transcriptional mechanisms by which RBM24 regulates early stages of embryonic myogenesis remain elusive. In C2C12 cells, RBM24 promotes myoblast fusion by inducing cell cycle arrest, but its targets are not clear. However, the highly related RBM38 exerts similar activity by directly stabilizing *p21* mRNA (Miyamoto et al., 2009). On the other hand, RBM24 binds to and stabilizes myogenin mRNA to induce the formation of myotubes (Jin et al., 2010). These results suggest that RBM24 may function to initiate myogenic differentiation by activating the translation of myogenic mRNAs. However, analysis of alternative splicing changes in *Rbm24* mutant mice also shows reduced inclusion of muscle-specific exons involved in muscle structure and functionality, suggesting that it is required for muscle terminal differentiation through regulation of muscle-specific alternative splicing (Yang et al., 2014). Consistently, RBM24 regulates similar muscle-specific alternative splicing events during differentiation of primary mouse satellite cells, in which down-regulation of RBM24 by miRNA-222 inhibits myoblast fusion (Cardinali et al., 2016).

Recent works indicate that a majority of muscle-specific splicing defects caused by loss of *Rbm24* in mice are also present in zebrafish *rbm24a* mutants, suggesting conserved mechanisms underlying RBM24 function in muscle development (Shao et al., 2020). However, the stability of several mRNAs encoding proteins critically involved in muscular function is also reduced in *rbm24a* mutants (Cheng et al., 2020), such as Smpx (small muscle protein X-linked) that functions to promote myocyte fusion by increasing the activity of NFAT (nuclear factor of activated T cells) and MEF2 transcription factors via IGF1 (insulin-like growth factor 1) signaling (Palmer et al., 2001). Smpx plays important roles in muscle fiber organization and distal myopathy (Ghilardi et al., 2021; Johari et al., 2021). This illustrates the functional implication of RBM24 in maintaining the stability and/or promoting the translation of muscle-specific mRNAs independently of alternative splicing. Consistently, RBM24 physically interacts with components of the cytoplasmic polyadenylation complex, including members of the cytoplasmic poly(A)-binding protein (PABPC) and the cytoplasmic polyadenylation element-binding protein (CPEB) families, which also show strong expression in the somites (Shao et al., 2020). Thus, RBM24 may be sequentially implicated in various post-transcriptional processes during myogenesis, which is likely dependent on its dynamic cytoplasm to nucleus translocation and on the differentiation state of muscle cells.

In adult mice, nuclear RBM24 is enriched in slow-twitch muscles along with myogenin (Grifone et al., 2021). Conditional knockout of *Rbm24* disrupts myoblast fusion and delays muscle regeneration by inhibiting muscle-specific alternative splicing events essentially as observed during embryonic myogenesis (Zhang et al., 2020). Further studies indicate that RBM24 also exhibits dynamic expression and activity during the muscle repair process, with the protein level rapidly increased in neoforming myofibers (Grifone et al., 2021). Knockdown of *Rbm24* in satellite cells impairs myogenin expression at early stages of muscle injury but affects muscle-specific splicing at late stages of regeneration, thereby preventing the differentiation of activated satellite cells into multinucleated myofibers (Grifone et al., 2021). Thus, RBM24 functions as a multifaceted regulator of muscle cell development and may be involved in maintaining the homeostasis of adult muscle. At present, although no mutation of the human *RBM24* gene has been directly associated with skeletal muscle disease, there is evidence that its expression is mis-regulated in DM muscular cells (Arandel et al., 2017; Wang et al., 2019), which may impair the proper function of its target genes and lead to defective muscle regeneration in diseased conditions.

### Tristetraprolin Family of Proteins Maintain Satellite Cell Homeostasis

Tristetraprolin (TTP), also known as zinc finger protein 36 homolog (ZFP36), is a founding member of the TPA-inducible sequence 11 (TIS11) family of RBPs. It binds to AREs present in target mRNAs through its two CCCH zinc-finger domains and promotes rapid mRNA decay. The other two members of the TIS11 family are ZFP36L1 (TIS11B) and ZFP36L2 (TIS11D). TTP may play a role in regulating satellite cell homeostasis and skeletal muscle regeneration as its expression is rapidly induced following muscle injury, before the activation of *MyoD* gene (Sachidanandan et al., 2002). Like STAU1 protein that maintains muscle stem cell quiescence by inhibiting *MyoD* mRNA translation (de Morrée et al., 2017), TTP promotes the decay of *MyoD* mRNA by binding to the 3′-UTR in mitotically dormant satellite cells, but it is rapidly phosphorylated and inactivated by p38α/β MAP kinase upon satellite cell activation, thus allowing the stabilization of *MyoD* mRNA and the differentiation of myofibers (Hausburg et al., 2015). ZFP36L1 and ZFP36L2 are also expressed in mouse satellite cells, with redundant function in maintaining the satellite cell population. Their simultaneous loss of function in PAX7-expressing satellite cells impairs muscle regeneration, resulting in decreased skeletal muscle mass and reduced body weight (Bye-A-Jee et al., 2018). In addition, the expression of ZFP36L1, but not TTP and ZFP36L2, is strongly induced during myogenic differentiation in C2C12 cells (Busse et al., 2008), but how it affects myogenic differentiation remains unclear. Thus, functional studies of TIS11 family members of RBPs in embryonic myogenesis are still lacking, and it would be of interest to determine their functional implication in myoblast proliferation and differentiation during early development.

### Other RNA-Binding Proteins Potentially Implicated in Diverse Aspects of Muscle Development

There are also many other RBPs that have been shown to play a role in embryonic myogenesis or skeletal muscle regeneration, although their post-transcriptional regulatory functions in myoblast proliferation and differentiation may be less extensively studied in comparison with the above-described RBPs. This section presents a non-exhaustive catalog of additional RBPs that are potentially implicated in muscle development and function in the embryo or in the adult.

ErbB3-binding protein 1 (EBP1) participates in ErbB3 receptor signaling. It is mainly accumulated in the nucleus of differentiating C2C12 myoblasts and activated mouse satellite cells, but preferentially localized in the cytoplasm of differentiated myotubes. Knockdown of *Ebp1* inhibits the proliferation and differentiation of C2C12 myoblasts and activated satellite cells (Figeac et al., 2014). In chick embryos, EBP1 is predominantly expressed in the epithelial dermomyotome and is required for myogenic differentiation of muscle progenitor cells (Figeac et al., 2014). Nevertheless, the role of EBP1 in embryonic myogenesis may be independent of its interaction with the ErbB3 receptor as the two proteins are expressed in distinct domains within the somite. Consistently, there is evidence that it promotes differentiation of chicken myoblasts through inhibition of SMAD2/3 signaling (Yu et al., 2017), but the exact post-transcriptional mechanism remains unclear.

IGF2 mRNA-binding protein 2 (IGF2BP2 or IMP2) may be important for myoblast growth and muscle regeneration independently of IGF2. It contains two RRMs and four KH domains, which can bind to various target transcripts. The expression of IGF2BP2 is increased in primary myoblasts and decreased in adult muscle, but it is re-activated during muscle regeneration (Boudoukha et al., 2010; Zhou et al., 2016). Knockdown of *Igf2bp2* in C2C12 myoblasts has been shown to decrease cell motility and migration capacity through up-regulation of PINCH2, a mediator of cell adhesion, and down-regulation of MURF3, a microtubule-stabilizing protein (Boudoukha et al., 2010). IGF2BP2 also promotes myoblast proliferation through up-regulation of N-RAS, MYC, MYF5, and cyclin A2, by increasing the stability or translation of their mRNAs (Li et al., 2012; Zhou et al., 2016). Consistent with its implication in the maintenance of myoblast proliferation, knockdown of *Igf2bp2* in C2C12 myoblasts and mouse satellite cells triggers myogenic differentiation (Zhou et al., 2016).

LIN28 is a highly conserved small cytoplasmic RBP regulating the transition from pluripotency to a differentiated cell fate (Tsialikas and Romer-Seibert, 2015). Its expression is strongly activated in differentiating myoblasts and in regenerating skeletal muscle fibers, but is barely detectable in proliferating myoblasts (Polesskaya et al., 2007). It functions as a positive factor of myogenesis through regulation of IGF2 expression by binding to its mRNA. In differentiating C2C12 myoblasts, LIN28 interacts with the translation initiation complex in an RNA-dependent manner and colocalizes with eukaryote initiation factor 3ß (eIF3ß) to enhance the translation of *Igf2* mRNA and promote myoblast differentiation (Polesskaya et al., 2007). However, further studies are needed to provide mechanistic insight into LIN28 function in embryonic myogenesis and adult skeletal muscle regeneration.

Quaking (QKI or QK) proteins are a family of RBPs with STAR (signal transduction and activation of RNA) domain and bind to AUCAA motifs in the 3′-UTR to regulate mRNA function. QKI-5 is predominantly localized in the nucleus, and its increased expression during myoblast differentiation regulates a large number of alternative splicing events involved in muscle function, among which the inclusion of muscle-specific exon from *Capzb* gene encoding a protein that regulates the growth of actin filament (Hall et al., 2013). QKI-5 also regulates the expression of Duchenne muscular dystrophy gene (*DMD*) in skeletal muscle by promoting the inclusion of its muscle-specific exon (Miro et al., 2020). Other QKI proteins may display cytoplasmic functions in fiber type specification. For example, zebrafish *qkia* is required for fast fiber maturation by regulating *gli2a* mRNA stability and translation (Lobbardi et al., 2011), while *qkia* and *qkic* functionally interact to control early myofibril assembly and sarcomere formation in slow muscle by regulating the accumulation of *tropomyosin-3.12* transcripts through binding to the 3′-UTR (Bonnet et al., 2017).

Transactivation response (TAR) RBP (TRBP) binds to double-stranded RNAs (dsRNAs). It acts as a regulator of miRNA pathway but also functions through miRNA-independent mechanisms. In C2C12 cells, TRBP regulates the expression of myogenic miRNAs, such as miR-1a and miR-133a, and is required for myoblast differentiation (Ding et al., 2016). Although mice with knockout of *Trbp* in skeletal muscle show no defective myogenesis, they display impaired muscle regeneration and increased fibrosis upon cardiotoxin-induced muscle injury, suggesting that it may play a role in promoting muscle regeneration and homeostasis (Ding et al., 2016). However, gene networks regulated by TRBP in the muscle repair process need further investigations.

DDX5 (p68) and its paralog DDX17 (p72) belong to a subfamily of DEAD-box RNA helicases that regulate multiple steps of RNA metabolism and display RNP chaperone activity (Xing et al., 2019). The expression of these RBPs decreases during differentiation of C2C12 cells (Dardenne et al., 2014). Interestingly, at early stages of myogenesis and at the beginning of EMT in cultured cells, they cooperate with hnRNP F/H to define myoblast-specific alternative splicing patterns, but upon induction of myoblast differentiation, they exert a differentiation-promoting activity by functioning as a partner and transcriptional coactivator of MYOD (Caretti et al., 2006; Dardenne et al., 2014). DDX5 may also modify RNA secondary structures and facilitate or stabilize MBNL1 binding to CUG repeats, thus contributing to the pathogenesis of DM1 (Laurent et al., 2012). The function of DDX5 and DDX17 in developmental myogenesis remains unknown, and it will be of interest to determine whether they regulate EMT during somite maturation. Another member of the DEAD-box family, DDX27, is required for skeletal muscle growth and regeneration during development. Mutation of *ddx27* in zebrafish leads to skeletal muscle hypotrophy and precocious skeletal muscle differentiation by disrupting ribosomal RNA maturation and thus the translation of a specific subset of transcripts during myogenesis (Bennett et al., 2018).

As the cold-inducible RNA-binding protein (CIRP), RBM3 is a highly conserved cold shock protein that shows transcriptional up-regulation in response to cold-stressed conditions (Zhu et al., 2016). In C2C12 myoblasts, overexpression of RBM3 is capable of counteracting the deteriorate effects of low temperature on myoblasts by preventing necrosis and by increasing viability, thus promoting survival of muscle cells (Ferry et al., 2011). RBM3 also functions as a potential regulator of skeletal muscle mass and may confer resistance to age- or disease-related muscle atrophy, because its increased expression in C2C12 myoblasts and in adult skeletal muscle enhances the formation of myotubes and myofibers, respectively (Van Pelt et al., 2018). Although RBM3 exerts multiple functions in the post-transcriptional regulation of gene expression, it plays a central role in muscle adaptive processes essentially by stabilizing target mRNAs and maintaining protein synthesis (Van Pelt et al., 2019).

Finally, the rising impact of post-transcriptional regulation on myogenesis is further illustrated by the unexpected observation that even MRFs, such as MYF5, can function as a RBP to promote mRNA translation. In proliferating and differentiating C2C12 myoblasts, MYF5 is localized in both the nucleus and the cytoplasm. It moderately activates the transcription of cyclin D1 gene but mostly enhances the translation of cyclin D1 mRNA by binding to the 3′-UTR and the coding region through the bHLH domain (Panda et al., 2016). These observations suggest that MYF5 regulates myoblast growth at the post-transcriptional level as well, in addition to its activity as a transcription factor.

## Functional Interactions Between RNA-Binding Proteins Coordinate Gene Expression During Myogenesis

**FIGURE 3:**
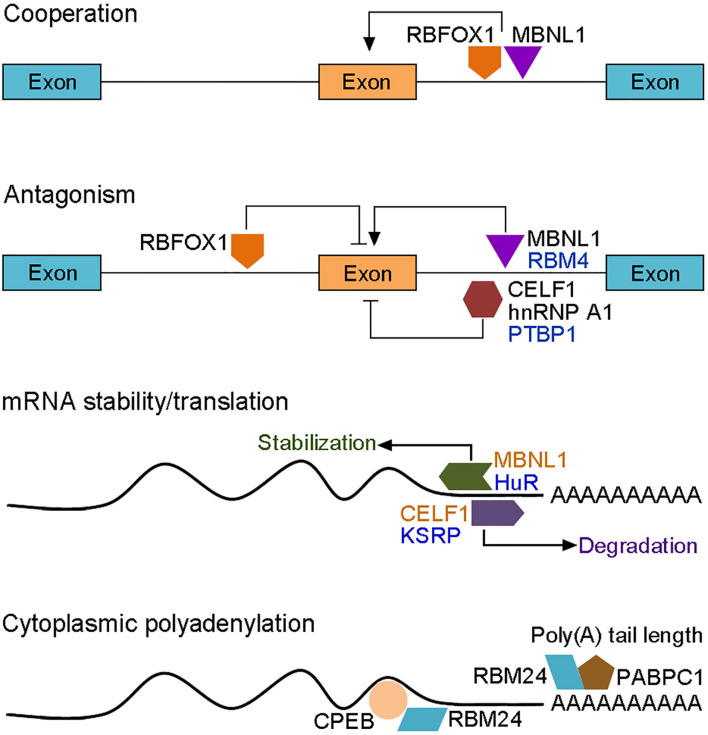
Functional interactions between RBPs in the post-transcriptional regulation of myogenic gene expression. RBPs (colored forms) act either cooperatively or antagonistically to control muscle-specific alternative splicing, cytoplasmic polyadenylation, mRNA stability and translation (arrows represent positive regulation; bars represent negative regulation; RBPs that display antagonistic activity in alternative splicing and mRNA stability are shown by identical colors). The activity of a particular RBP is also dependent on its interaction with the pre-mRNA. RBFOX1 inhibits or promotes inclusion of the adjacent exon upon upstream or downstream binding, respectively. RBPs-mediated mRNA degradation or translation either positively or negatively regulates myogenic differentiation, depending on the targets. RBM24-mediated cytoplasmic polyadenylation of muscle-specific target mRNAs also await further investigation.

Transcriptome and proteome studies have not only identified hundreds of muscle-specific RBPs but also revealed changes in the interactome during muscle differentiation (Hiller et al., 2020). With so many RBPs dynamically implicated in different steps of myogenesis and in multiple aspects of post-transcriptional regulation of gene expression, it is not surprising that they functionally interact in a tight network to coordinate muscle development and maintain muscle homeostasis. Thus RBPs are multifaceted regulators that function either cooperatively or antagonistically to orchestrate the myogenic program during embryonic and adult myogenesis. The modes and outcomes of their interactions are complex and context-dependent. Here we present a few better-described examples to illustrate the interplay between RBPs in different aspects of post-transcriptional regulation (Figure 3). Interference with RBFOX1 or MBNL1 function in normal embryonic muscle cells indicates that they co-regulate a common subset of muscle-specific alternative splicing events with either inclusion or exclusion of exons, depending on upstream or downstream binding of RBFOX1 to the pre-mRNA (Conboy, 2017). This raises the possibility that the combined loss of their activity in DM1 may further compromise the expression of genes involved in muscle function (Klinck et al., 2014). However, RBFOX1 can also compete with MBNL1 for binding to CCUG repeats in myoblasts, thus partially correcting alternative splicing defects of *CLCN1* and to some extent rescuing muscle atrophy (Sellier et al., 2018). As highlighted above, functional antagonism clearly exists between CELF1 and MBNL1 in regulating mRNA localization, stabilization and translation (Wang et al., 2015). CELF1 binds to the 3′-UTR of mRNAs and promotes destabilization, while MBNL1 binds to other regions of the same 3′-UTR to promote stabilization (Vlasova and Bohjanen, 2008; Wang et al., 2012). The two proteins also function as antagonistic regulators of alternative splicing in DM1 (Wang et al., 2015), with gain of CELF1 and loss of MBNL1 collectively contributing to the reversion of fetal-specific splicing patterns in adult skeletal muscle. As CELF1, hnRNP A1 antagonizes the activity of MBNL1 in DM1 muscle by promoting the inclusion of fetal exons, but the exclusion of adult exons (Li et al., 2020). Similarly, there is also a widespread antagonism between CELF2 and RBFOX2 in regulating the expression of muscle-specific protein isoforms, such as members of the MEF2 family (Gazzara et al., 2017). The cooperation or antagonism between RBPs helps to fine-tune each step of myogenesis, thus unbalanced interactions lead to defective muscle development and occurrence of muscle disease.

**FIGURE 4:**
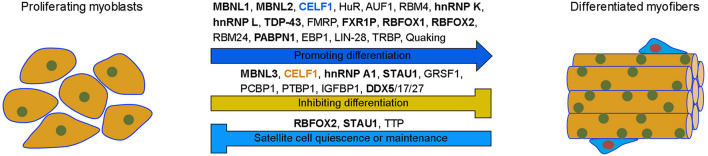
Differential functions of RBPs in myogenesis. Many RBPs promote the differentiation of proliferating myoblasts into functional myofibers, while others either inhibit myogenic differentiation or maintain satellite cell quiescence. Note that cytoplasmic CELF1 (blue) promotes, while nuclear CELF1 (orange) inhibits, muscle differentiation by regulating the stability of myogenic mRNAs or by repressing the inclusion of muscle-specific exons, respectively. RBPs in bold indicate their potential implication in muscle disease, such as MD, FSHD, or OPMD.

In addition to these cross-regulatory interactions, there exists also expression antagonism between RBPs during myogenesis. This is important for RBPs to function at the right time and place in muscle cells. For example, *PABPN1* mRNA is highly expressed in primary myoblasts but its stability is strongly reduced in mature muscle, resulting in reduced protein expression (Apponi et al., 2013). HuR may be involved in regulating the decay of *PABPN1* mRNA by binding to AREs within its 3’-UTR, thus preventing its translation at the end of myogenesis, suggesting that HuR functions as a negative regulator of PABPN1 expression to control its activity in adult muscles (Phillips et al., 2018). Similarly, PTBP1 expression level is strongly reduced during C2C12 myoblast differentiation (Boutz et al., 2007). This suppression in differentiated muscle cells may involve RBM4-mediated alternative exon skipping of *Ptbp1* transcripts, indicating that RBM4 promotes myogenesis in part by antagonizing the activity of PTBP1 through down-regulation of its expression (Lin and Tarn, 2011). Therefore, RBPs display both distinct and redundant functions in muscle biology. While some of them promote myogenesis either *in vivo* or *in vitro*, others function to inhibit myogenic differentiation or maintain stem cell quiescence (Figure 4).

RNA-binding proteins also physically interact and form complexes to regulate gene expression in myogenic cells. KSRP is an RBP that serves as an mRNA decay-promoting factor. During muscle differentiation, it is phosphorylated by p38 MAP kinase, preventing its interaction with myogenic transcripts and its mRNA destabilizing function (Briata et al., 2005). In undifferentiated muscle cells, the C-terminus of HuR mediates the formation of the HuR-KSRP complex, which destabilizes nucleophosmin and *Ppargc1a* mRNAs encoding a regulator of cell cycle progression and a coactivator of cellular energy metabolism, respectively, thus promoting the commitment of muscle cells during early steps of myogenesis (Cammas et al., 2014; Janice Sánchez et al., 2019). DDX5 may directly interact with hnRNP F/H and facilitate its binding to highly structured G-quadruplex-enriched regions, thus initiating myoblast-specific splicing programs and regulating myogenic differentiation (Dardenne et al., 2014). In DM1 muscles, hnRNP L may regulate the same targets as MBNL1, which is consistent with the their physical interaction (Alexander et al., 2021). It is also of note that RBM24 interacts with several RBPs of the cytoplasmic polyadenylation complex, including PABPC1 and CPEB4, to regulate poly(A) tail length (Shao et al., 2020). Given the important function of RBM24 in muscle differentiation and regeneration, it will be of interest to examine how this interaction regulates the stability and translation of muscle-specific mRNAs. Therefore, further investigations of the complex interplay between RBPs should shed light on the gene regulatory networks orchestrating muscle development and homeostasis.

## Concluding Remarks

The RBP world is expanding rapidly with proteome-wide identification of proteins involved in RNA binding and function (Hentze et al., 2018). It is clear that RBPs function sequentially to post-transcriptionally regulate gene expression at different steps of myogenesis, in much the similar manner as different myogenic transcription factors that promote myoblast proliferation, myogenic differentiation and satellite cell quiescence at the transcription level. Given the growing number of muscular disorders associated with RNAs and RBPs, understanding the functional implication of RBPs in different steps of muscle development may have the potential to use them as therapeutics (Shotwell et al., 2020). However, there are also many fundamental questions that remain open for further investigations.

At present, muscle functions of RBPs have been mostly studied in diseased conditions or in cultured cell lines, while their regulatory roles during embryonic myogenesis and adult skeletal muscle regeneration remain largely elusive. Many RBPs display dynamic expression and subcellular localization during skeletal muscle differentiation, while inappropriate regulation of their expression and cytoplasm to nucleus or nucleus to cytoplasm translocation contributes to muscle disease. Using appropriate animal models such as the transparent zebrafish embryos, CRISPR/Cas9-mediated gene-tagging approach combined with time-lapse live imaging could help to define the dynamic characteristics of RBPs subcellular partitioning during embryonic myogenesis and their spatiotemporal activation in neoforming myofibers during muscle regeneration. Besides the importance to decipher the mechanism underlying RBP shuttling between different cellular compartments, which should provide insight into the regulation of myoblast proliferation and differentiation, it is also of interest to understand the nuclear versus cytoplasmic function of RBPs during normal myogenic differentiation and in diseased conditions. A close related intriguing question is the dynamic and distinct functions of RBPs in the proliferation or differentiation of muscle cells. Several RBPs, such as HuR and RBM24, are first localized in the cytoplasm of myoblasts to regulate mRNA stability and then accumulated in the nucleus in matured muscles to regulate alternative splicing. These characteristics may be regulated by the formation of protein-protein and RNA-protein complexes, but the identity of protein partners and mRNA targets for different RBPs remains to be characterized in future study. Moreover, it is important to elucidate how functional interactions, either cooperative or antagonistic, contribute to fine-tune target gene expression patterns and myogenic networks. There is also mutually exclusive expression between RBPs, with some being expressed in early steps of myogenesis, while other being activated in differentiated muscles. These differential associations of RBPs in myoblast and myotube interactomes regulate different steps of myogenesis.

The expression and activity of different RBPs are tightly controlled in mature myofibers under steady state as well as in stressed conditions. Accordingly, RBPs stabilizing myogenic mRNAs are rapidly up-regulated while splicing factors are down-regulated during satellite cell activation (Farina et al., 2012). This critically contributes to the maintenance of muscle homeostasis and satellite cell quiescence or activation. Imbalance of either their early or late function leads to muscle atrophy. Another important aspect that is briefly introduced in this review concerns the functional interactions between RBPs and non-coding RNAs, such as miRNAs, circRNAs, and IncRNAs. Their mutual regulations have either positive or negative impact on myogenic gene expression, further adding complexity to the control of developmental myogenesis and progression of muscle disease. Moreover, even MRFs can get involved in mRNA translation, thus the implication of post-transcriptional regulation in muscle biology will continue to provide surprises and breakthroughs in the future. System-wide identification of interactome in muscle cells can advance our knowledge of RBP functional interactions. Bioinformatic analyses combined with experimental validations of RBPs-regulated networks should provide a better understanding of post-transcriptional mechanisms underlying myogenic differentiation and muscle disease.

## Author Contributions

D-LS and RG performed literature analyses and prepared figures. D-LS wrote the manuscript. D-LS and RG reviewed and edited the final version of the manuscript. Both authors contributed to the article and approved the submitted version.

## Conflict of Interest

The authors declare that the research was conducted in the absence of any commercial or financial relationships that could be construed as a potential conflict of interest.

## Publisher’s Note

All claims expressed in this article are solely those of the authors and do not necessarily represent those of their affiliated organizations, or those of the publisher, the editors and the reviewers. Any product that may be evaluated in this article, or claim that may be made by its manufacturer, is not guaranteed or endorsed by the publisher.

## Footnotes

1
www.ensembl.org
